# Optimizing Flaxseed Oilcake Yield and Nutritional Quality Through Sowing Density and Fertilization Management

**DOI:** 10.3390/plants15213358

**Published:** 2026-10-29

**Authors:** Panteleimon Stavropoulos, Antonios Mavroeidis, Stella Karydogianni, Ioannis Roussis, Stavroula Kallergi, Myrto Chatzitriantafyllou, Eleni Ntzoumani, Eleni Tsiplakou, Ioanna Kakabouki

**Affiliations:** 1 Laboratory of Agronomy, Department of Crop Science, Agricultural University of Athens, 11855 Athens, Greece; antoniosmauroeidis@gmail.com (A.M.); karydogianni@aua.gr (S.K.); roussis@aua.gr (I.R.); kallergi@aua.gr (S.K.); myrtochatz16@gmail.com (M.C.); ntzoumanieleni@gmail.com (E.N.); 2 Laboratory of Nutritional Physiology and Feeding, Faculty of Animal Science, Agricultural University of Athens, 11855 Athens, Greece; eltsiplakou@aua.gr

**Keywords:** animal feed, density, fertilization, Flax (*Linum usitatissimum* L.), oilcake, nutritional value

## Abstract

Flaxseed oilcake, the by-product of oil extraction, has potential as a feed ingredient. The effect of agronomical practices on the oilcake remains limited. This study evaluated the effect of sowing density and type of fertilizer on oilcake yield and nutritional value. A two-year field experiment was conducted in a split-plot design with two sowing densities (500 and 300 plants m^−2^) and four fertilization treatments: control, urea, urea with urease and nitrification inhibitors, and an organic fertilizer (compost and tomato pomace). Oilcake was obtained by cold pressing and analyzed for dry matter, ash, crude protein, fat, crude fiber, neutral detergent fiber (NDF), and acid detergent fiber (ADF), while feeding indices were estimated using predictive equations. Year did not affect any trait, whereas the interaction between sowing density and fertilizer type affected most traits. Urea with inhibitors produced the highest oilcake yield at the low density (42% higher than the control), whereas at the high density it did not differ from the control. Fertilization increased protein content by 5–12%, while urea with inhibitors increased ash and, under high density, produced the highest protein and fat contents, but also increased NDF. Crop management thus affected both the quantity and composition of flaxseed oilcake, with trade-offs between yield and compositional traits.

## 1. Introduction

Agro-industrial by-products could be a promising alternative feed ingredient, leading to a more sustainable and circular agri-food system [1]. Under that context, oilcakes, the by-product remaining after oil extraction, could be a valuable animal feed ingredient, due to its high nutritional value [2]. Flaxseed (*Linum usitatissimum* L.) has raised interest by its favorable nutritional profile [3], characterized by high levels of protein, dietary fiber and oil, rich in α-linolenic acid (ALA) [2,4]. Flaxseed oilcake has high crude protein content (28–38%) [5], oil rich in ALA which remains after the extraction, fibers, minerals, lignans and phenolic compounds [4], which increase its nutritional and functional value as an ingredient in animal feed [6,7]. The use of flaxseed oilcake in animal diet has been reported that improves animal performance and antioxidant status, in different animal categories, including ruminants, pigs, poultry and fish, while the ALA content seems to contribute to enriching animal-derived products with n-3 polyunsaturated fatty acids [8,9,10,11,12,13].

Oilcake’s nutritional value is correlated with seed’s chemical composition, which is affected by agricultural practices. Sowing density and fertilizer type are two key factors, influencing crop growth and seed quality, and may therefore affect the nutritional value of oilcake [14,15]. According to other studies, the type of fertilizer, combined with different agricultural practices, can affect the nutritional value of oilcake in *Camelina sativa* (L.) Crantz, highlighting the importance of these factors for the high quality of feed products [16]. Nevertheless, the combined effects of sowing density and fertilization on the nutritional quality of flaxseed oilcake remain insufficiently investigated. In addition, the use of organic fertilizer, made by tomato pomace (a by-product from the industrial process of tomato), is also limited in the literature, and could lead to the production of higher-value feed ingredients.

The present study aimed to evaluate the combined effects of sowing density and fertilization strategy, including the application of an organic fertilizer derived from tomato-processing residues, on the nutritional quality of flaxseed oilcake.

## 2. Results

The combined analysis showed that the factor “year” and its interaction with sowing density, or fertilizer did not statistically significantly affect the results of the study. According to this, results are presented as means across the two experimental periods.

### 2.1. Oilcake Yield and Recovery

**Figure 1:**
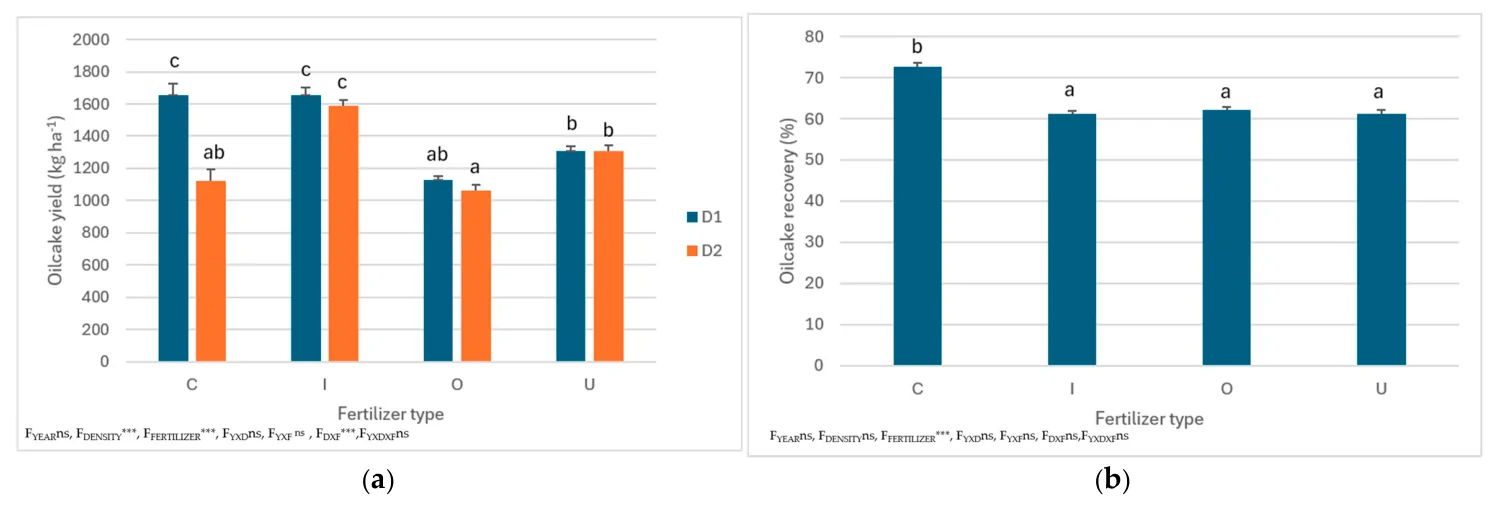
Effects of sowing density and fertilizer type on oilcake yield (**a**) and oilcake recovery (**b**). Sowing density included D1 (500 plants m^−2^) and D2 (300 plants m^−2^). Fertilization treatments included C (control), U (urea), I (urea with urease and nitrification inhibitors), and O (organic fertilizer). Values are means ± SE across two years presented for each density × fertilizer combination in (**a**) (*n* = 8) and for each fertilizer type across densities in (**b**) (*n* = 16). Different letters indicate statistically significant differences among the presented means, according to Tukey’s HSD test (*p* < 0.05), using the split-plot error terms. The symbols “ns” and “***” indicate non-significant effects and significance at *p* < 0.001, respectively.

Oilcake yield was affected by sowing density, fertilizer type and the interaction between them. In high density (D1), the highest yield was reported in I and C. The differences between these two treatments were non-significant. In U and O, oilcake yield was 21 and 32% lower, respectively, compared to C. At low density, the use of I led to the highest yield, 42% higher than the control, whereas U and O did not statistically significantly differ from the control. Oilcake recovery refers to the percentage of oilcake produced from the pressing of the seeds. In this study, this percentage was highest in C, whilst the others did not differ statistically significantly from one another. More specifically, the treatments that received fertilizer recorded values approximately 10–11 percentage points lower than the control (Figure 1).

### 2.2. Nutritional Value

Dry matter content of the oilcake was statistically significantly affected by the interaction between sowing density and fertilization type. The highest dry matter content was recorded in treatment D2O, whilst the lowest was in D2C. In addition, ash content was statistically significantly affected by the interaction between sowing density and fertilization type. The highest ash content was recorded in I at both densities, which did not differ statistically significantly from each other. The lowest value was recorded in treatment D1C. In D1, the use of the inhibited fertilizer increased ash content in the oilcake by 19%, followed by urea (8%) and organic fertilizer (6%), compared with C. In D2, ash content increased by 6 and 2% in I and O, respectively, but decreased by 11% in U, compared to C. Protein content was statistically significantly different between the interaction of sowing density and fertilizer type. Fertilization increased CP compared with C by 6–12% in D1 and 5–9% in D2. The lowest values were recorded in treatments D1C and D2C, although there was no statistically significant difference between these two. The highest protein content was recorded in treatment D1I, which did not statistically significantly differ from D1O (Table 1). Statistically significant differences were observed in the interaction between sowing density and fertilization type for the fat content too. The lowest values were recorded in D2C, whilst the highest were recorded in D1I. The use of urea with nitrification and urease inhibitors, combined with a higher sowing density, led to a 44% increase in the oilcake’s fat content, compared to D2C. In D1, the use of fertilizer increased fat content by 29%, 16% and 5% for I, O and U, respectively, compared with D1C. In D2 I, O and U were reported to be 16%, 5% and 5% higher compared with D2C. Between densities, D1C, D1I, D1O and D1U were 11%, 23%, 24% and 12% higher compared to those in D2 (Table 1).

**Figure 2:**
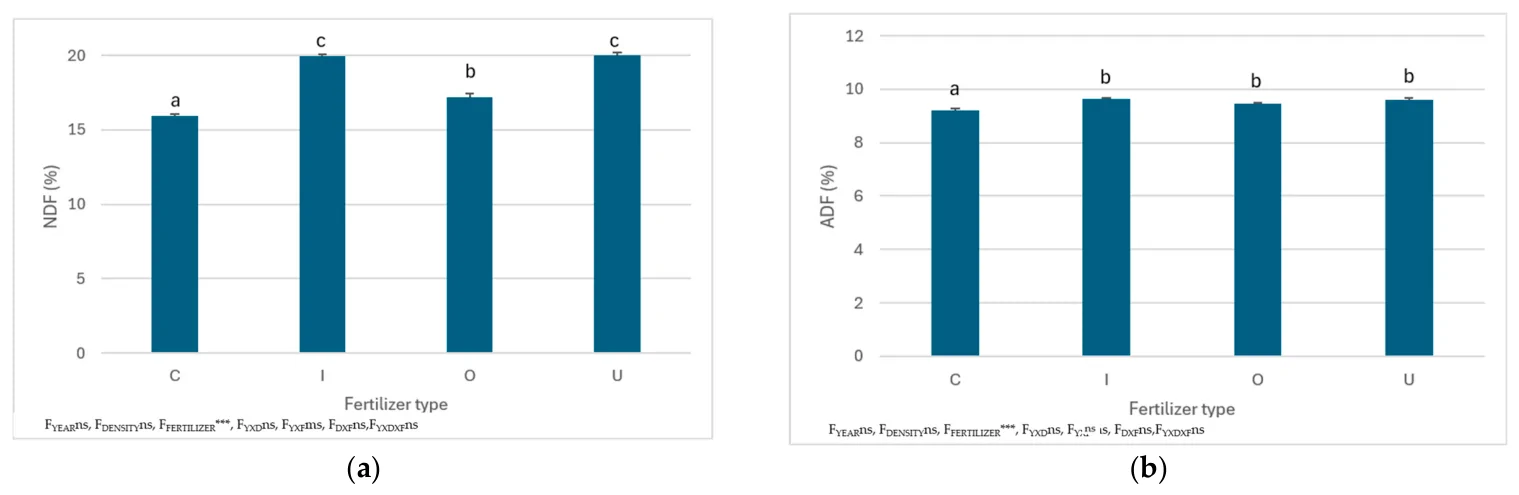
Figures represent the effect of four fertilizers (C—control, U—urea, I—inhibited urea and O—organic fertilizer) on NDF (**a**) and ADF (**b**) content. Values are means ± SE across sowing densities and years (*n* = 16). Different letters represent statistically significant differences between fertilizer types (Tukey’s HSD, *p* < 0.05). The symbol “ns” indicates non-statistically significant differences, while “***” indicates significance at the *p* < 0.001 level.

Fiber content was also statistically significantly affected by the interaction between sowing density and fertilizer type, with D1I, D1O and D2I reporting the highest values for this trait. The lowest fiber values were recorded in D1U, which did not statistically significantly differ from D2O (Table 2). At D1, the use of I and O increased fiber content, compared with C, while U decreased it. At D2, I and U increased fiber content, whereas O did not report a statistically significant difference from C. NDF and ADF were affected only by fertilization type. NDF was highest under U and I and lowest in C. All fertilizers increased ADF compared with C, with no significant differences among them (Figure 2).

### 2.3. Estimated Feeding Indices

The multifactor analysis showed that the estimated metabolizable energy (ME) for ruminants and pigs, as well as TDN, were statistically significantly affected by the interaction between sowing density and fertilizer type (Table 3). Estimated MEpigs was also highest in D1I, while U reported the lowest values for both D1 and D2. In D2 C, I and O did not statistically significantly differ from each other. Estimated MEruminants and TDN were statistically significantly highest in D1U and D2O, and lowest in D2I. Estimated DDM, DMI and RFV were statistically significantly affected only by fertilizer type. DMI and RFV were highest in C and lowest in I and U. Estimated DDM was higher in C and lower in all fertilized treatments, with no statistically significant differences between them (Table 4).

## 3. Discussion

Dry matter and ash concentration in the oilcake varied according to the combined effect of fertilization and sowing density. These differences may be associated with changes in seed biomass accumulation and with shifts in the contribution of the different seed constituents that remain in the solid fraction following oil extraction (oilcake) [2]. From a feed-quality perspective, a high dry matter concentration is advantageous because it corresponds to a greater nutrient concentration and reduces the contribution of moisture to the overall mass [15]. Urea with nitrification and urease inhibitors resulted in the highest ash concentration at both densities, whereas the response to conventional urea and organic fertilization depended on sowing density. This response may be related to enhanced mineral nutrient uptake after fertilizer application and subsequent accumulation of these elements in the developing seeds [17].

Oilcake yield was significantly affected by the interaction between sowing density and fertilizer type. The yield of oilcake reflects both the influence of the studied factors on seed production and the proportion of oilcake remaining after the extraction process. The oilcake recovery was approximately 10–11 percentage points lower in the fertilized treatments compared to the control. Nitrogen fertilization has been reported to increase the oil content of the seeds [18], which in turn reduces the proportion of oilcake. Consequently, control exhibited high values for both oilcake yield and oilcake recovery. Urea with urease and nitrification inhibitors also showed similarly high yield values. This is likely due to the slower release of nitrogen, which extended its presence in the soil and improved synchronization with the crop’s requirements. Nitrogen inhibitors can reduce nutrient losses through leaching and volatilization, ensuring that nitrogen remains available during critical stages of crop development [19]. It has been reported that the use of urea with urease and nitrification inhibitors can improve crop’s yield [20,21]. Given that all treatments involving fertilization received the same total amount of N, the lower oilcake yield observed with organic fertilization is likely due to reduced availability of nitrogen to the plants, as only a portion of the organic nitrogen is mineralized during the growing season [22].

Crude protein concentration was determined by the interaction between fertilizer treatment and sowing density, demonstrating that protein accumulation depended on both nitrogen management and crop growth conditions. Fertilization increased crude protein at both densities, with the highest value recorded under urea with urease and nitrification inhibitors at the high sowing density, which did not differ from organic fertilization at the same density. The prolonged availability of nitrogen during the later stages of vegetative development may have increased amino acid synthesis, providing the substrates required for protein formation. Greater nitrogen availability during seed filling has been associated with enhanced nitrogen accumulation in the seed [23], which may ultimately increase crude protein concentration in the residual oilcake. Organic fertilization produced similarly high protein levels to those obtained with inhibited urea. This may be attributed to the progressive mineralization of organic nitrogen, which may support a more continuous nutrient supply during seed filling. Maintaining nitrogen availability during the final stages of development is particularly important for protein synthesis because the proteins accumulated in the seed remain largely in the oilcake after oil extraction [16].

Fat concentration was also affected by the interaction between fertilization and sowing density, with the maximum value recorded under high sowing density combined with urea containing inhibitors. The residual fat content of the oilcake reflects both the oil content of the seed and the efficiency of oil extraction during pressing. The lower oilcake recovery observed in the fertilized treatments suggests that their seeds contained more oil, part of which remained in the oilcake. At the same time, the relatively high residual fat of all oilcakes (17–24%) indicates the limited extraction efficiency of the small-scale screw press, which may also have differed among seed lots; therefore, the observed differences cannot be attributed exclusively to seed oil synthesis. Improved nitrogen-use efficiency under inhibitor application may promote fatty-acid formation within the developing seeds [23]. Adequate nitrogen supply can increase photosynthetic activity and consequently enhance the availability of assimilates and carbon skeletons required for fatty-acid biosynthesis [16]. By limiting nitrogen losses, inhibitors may therefore maintain more favorable nutritional conditions during seed filling, resulting in greater fatty-acid accumulation and a higher fat concentration in the oilcake. This characteristic is particularly relevant when linseed oilcake is considered as an animal-feed ingredient because its lipid fraction contributes to its nutritional value and energy density [24]. However, the high proportion of unsaturated fatty acids makes careful processing and storage necessary to minimize oxidative deterioration and maintain product quality [25]. In the present study, this risk was limited by storing the oilcake samples in a proper way.

The fiber profile, represented by NDF and ADF, responded mainly to fertilization. Urea and urea with urease and nitrification inhibitors reported the highest NDF concentrations, whereas all fertilizer treatments increased ADF to a similar extent compared with the control. The greater accumulation of structural fractions following mineral fertilization may be related to more vigorous plant growth and increased synthesis of structural carbohydrates [26]. Greater nitrogen availability may have stimulated photosynthesis and biomass production [21], while also contributing to the formation of cell-wall constituents such as cellulose and hemicellulose. NDF is composed mainly of cellulose, hemicellulose and lignin, and is related to potential dry matter intake, whereas ADF contains mainly cellulose and lignin and is more closely associated with feed digestibility [27,28,29]. Consequently, higher NDF and ADF indicate a greater contribution of structural carbohydrates, which can support normal digestive function, particularly in ruminants, although excessive fiber concentrations may restrict intake and digestibility. In the present study, ADF changed relatively little compared with NDF. This pattern suggests that the main effect of fertilization was probably associated with the overall cell-wall fraction, with a greater contribution from hemicellulose than from the less digestible fraction represented by ADF. As DMI, DDM and RFV are calculated directly from NDF and ADF (Section 4.3), their differences among treatments mirror the responses of the fiber fractions and do not provide independent evidence of intake or digestibility; therefore, they are not interpreted further.

The feed evaluation of the oilcake further showed that agronomic treatments modified the estimated energy indices, which were affected by the interaction between sowing density and fertilization. Thus, the feeding value of the oilcake appears to depend on the combined influence of these two factors on seed composition and, subsequently, on the composition of the extracted oilcake. Estimated pig ME was highest in D1I, mainly due to its high fat content. However, the pig ME equation was developed for flaxseed expellers with lower fat content than the present oilcakes [30]; therefore, the absolute values are likely overestimated and are useful only for comparing treatments. TDN and ruminant ME varied little among treatments, because fertilization increased both crude protein, which increases TDN in the equation used [31], and crude fiber, which decreases it. As this equation does not include fat, the higher fat content of some oilcakes is not reflected in these estimates.

To summarize the results of the study, the use of urea with urease and nitrification inhibitors resulted in high oilcake yield in both densities and the highest protein and fat content at the high density. However, this treatment also increased NDF, which resulted in lower estimated DMI and RFV. Organic fertilization reported a protein level similar to those of inhibited urea and intermediate NDF values, but a lower oilcake yield. These trade-offs indicate that the choice of fertilization strategy depends on whether oilcake quantity or specific compositional traits are prioritized.

Despite the promising findings regarding the potential nutritional value of flaxseed oilcake as animal feed, it should be acknowledged that the nutritional indices employed in the present study are based on predictive equations, and therefore represent estimates rather than direct measurements of animal responses. Consequently, further research involving controlled feeding trials is needed to validate these findings and evaluate the effects of incorporating flaxseed oilcake into practical animal diets on nutrient utilization, digestibility, and animal performance. Despite the high nutritional value of oilcake, it is important to note that studies have identified that it contains toxic substances, such as cyanogenic glycosides [32]. It is worth noting that the concentration of these substances depends on the seed pressing process and the method of oilcake production, whilst any harmful effects also depend on the species of animal [33,34]. Although previous studies have reported that flaxseed can be included in diets of certain animal species at moderate levels without adverse effects [33], cyanogenic glycosides, appropriate inclusion rates and animal responses were not assessed in the present study. Further research, including the determination of cyanogenic glycosides, is therefore needed to evaluate these compounds and the effect of agronomic practices on their concentration.

## 4. Materials and Methods

### 4.1. Crop Establishment

**Figure 3:**
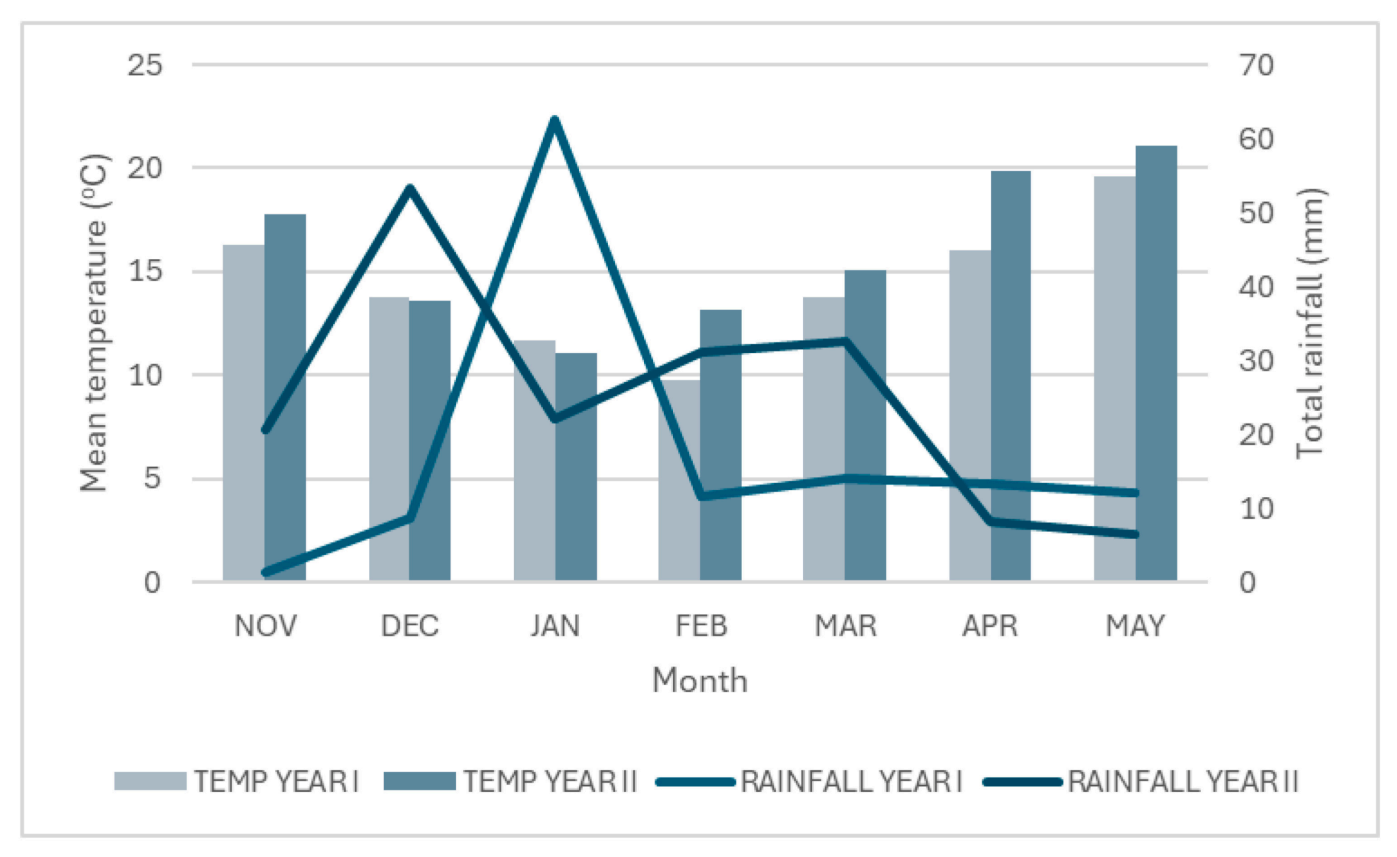
Meteorological (mean temperature [°C] and total precipitation [mm]) data during experimental periods. The first period, year I, refers to November 2022 to May 2023, while year II refers to November 2023 to May 2024.

A field experiment was laid out at the Agricultural University of Athens (37°59′ N and 23°42′ E; 30 m altitude), for two experimental periods (November 2022–May 2023 [year I], and November 2023–May 2024 [year II]), in an area of 837 m^2^. The soil at the experimental field was clay loam, with a pH of 7.39 (1:1 H2O) and soil organic matter (SOM) 1.67%, total nitrogen 0.143%, calcium carbonate (CaCO3) 15.34%, phosphorus (Olsen P) 13.6 mg kg^−1^ and potassium (K) 233 mg kg^−1^. The meteorological data were collected daily, using a weather station (Davis Vantage Pro2 Weather Station; Davis Instruments Corporation, Hayward, CA, USA), which is established in the experimental field. During the experimental period, the mean temperature was 14.4 and 16.0 °C for years I and II, respectively, while total rainfall was reported 123.7 and 174.0 mm (Figure 3).

**Figure 4:**
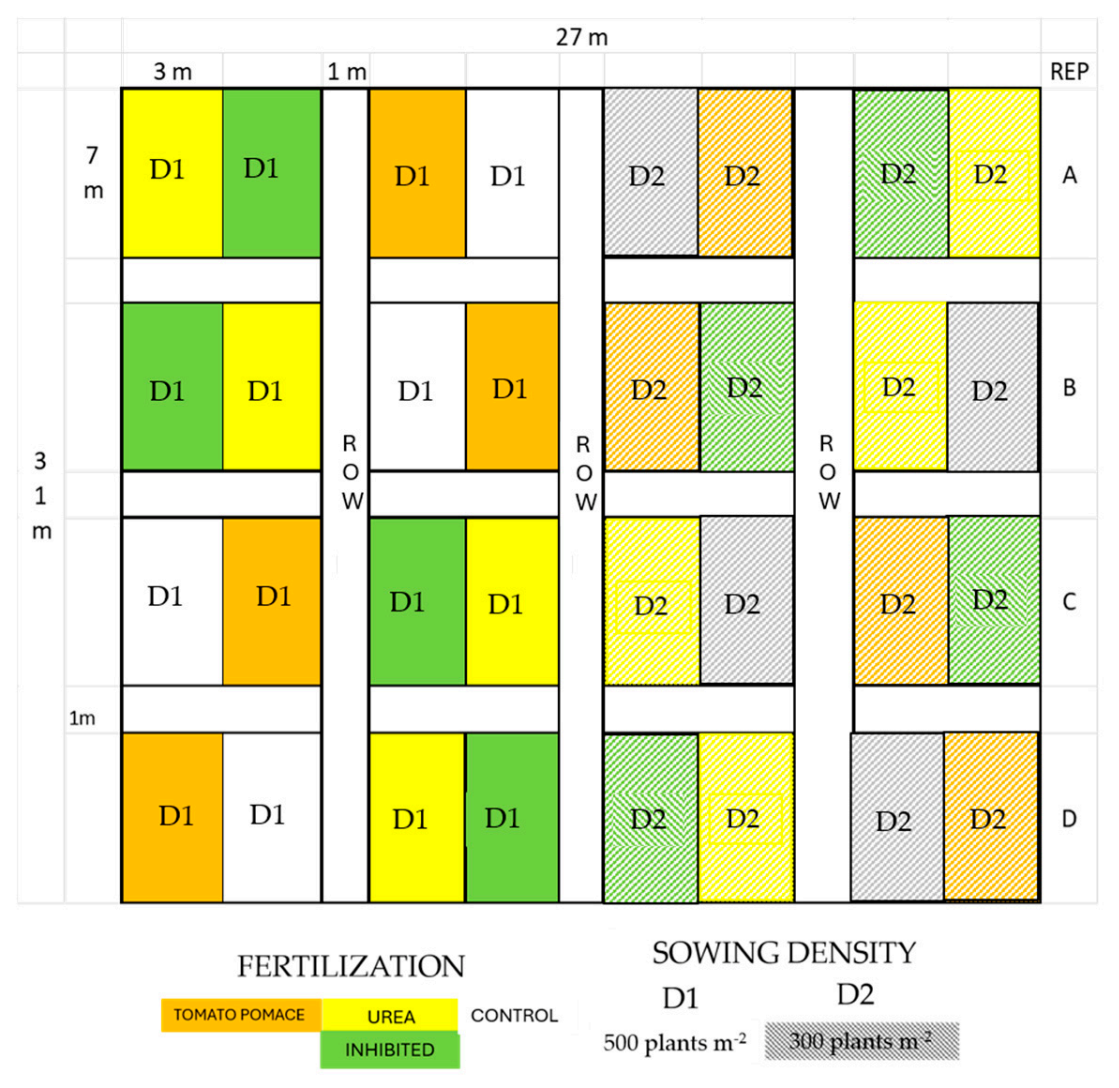
Field layout arranged in a split-plot design with four replications. Sowing density (D1: 500 plants m^−2^ and D2: 300 plants m^−2^) was assigned to the main plots, while four fertilization treatments (control, urea, inhibited urea, and organic fertilizer) were randomly allocated to the sub-plots. Different colors indicate fertilization treatments, and hatched patterns distinguish the D2 sowing density.

Two factors were evaluated in four replicates, using a split-plot design. The main plot (91 m^2^—13 m × 7 m) referred to sowing density, with 500 plants m^−2^ (D1) and 300 plants m^−2^ (D2) for high and low density, respectively. The sub-plot (21 m^2^—3 m × 7 m) was fertilizer type. Four treatments were evaluated with an unfertilized control (C), conventional urea (U), urea with inhibitors (I) and an organic fertilizer (O). Within each block, sowing densities were randomly allocated to the main plots, while fertilizers were also randomly allocated to the sub-plots, within each main plot. The actual field layout resulting from this randomization is presented in Figure 4. The same layout was used in both seasons; second-year results may therefore include residual effects of the first-year fertilization. All fertilizers were applied before sowing and incorporated into the soil. The application rate was in accordance with the mean nitrogen (N) rate recommended by the literature (80 kg N ha^−1^) [3]. The main characteristics of the fertilizers are presented in Table 5.

Flax (*Linum usitatissimum* L. cv Everest) seeds were sown in rows. The distance between rows was 25 cm for D1 and 35 cm for D2. Sowing was performed by hand, with a seeding rate higher than that of the target population. After emergence, seedlings were thinned to 125 and 100 plants per meter of row (12 and 9 rows per sub-plot), respectively, in order to establish the target densities. The crop was rainfed. Weed management was performed weekly by hand, while no infestations were reported during the experimental period. Sowing took place on 25 November 2022 and on 25 November 2023, while harvest took place on 9 May 2023 and 10 May 2024. The crop was harvested by hand, with a seed moisture at 12%.

### 4.2. Measurements

Seed yield was determined by hand-harvesting each sub-plot (21 m^2^) at 12% seed moisture. After harvest, seeds were air-dried at room temperature in a protected environment until seed moisture reached 8%. For the oilcake samples, 300 g of harvested seeds per plot were weighed and cold pressed separately via a mechanical pressing machine (TäbyPress Type 20, Örebro, Sweden), without external heat. The press was thoroughly cleaned after each pressing and allowed to cool totally between samples, preventing heat build-up during operation. Oilcake samples were left to dry at room temperature for 24 h and weighed. Oilcake recovery (%) was calculated as the mass of oilcake obtained from 300 g of seed × 100/300, and oilcake yield (kg ha^−1^) as seed yield × oilcake recovery/100. Subsequently, the samples were stored in airtight containers at 4 °C, in a dark environment protected from direct sunlight, for approximately 72 h until analyses.

For the quality analysis, the samples were ground using a 1 mm Wiley mill (Thomas T4274.E15 Steel Model 4 Wiley Mill; Arthur H. Thomas. Philadelphia, PA, USA). The analysis for the quality traits of oilcake was performed according to the Association of Official Analytical Chemists [35] and are presented below (Table 6). Each sample was analyzed in duplicate, and the mean of the two determinations was used. Prior to fiber analyses, samples were defatted by immersion in petroleum ether for 10 min. NDF and ADF residues were ashed to express both fractions exclusive of residual ash. All results are expressed on a dry-matter basis.

### 4.3. Evaluation as Animal Feed

The evaluation of oilcake as animal feed was based on the use of indices. These indices are presented below. These equations were developed for specific categories of feed, the ME equation for pigs in relation to flaxseed meal residues [30], the TDN equation for ruminant feed [31], and the DMI, DDM and RFV equations for forage [38,39]. Their application in this particular study, which concerns a high-fat oilcake, a by-product of cold pressing, represents an extension beyond their original calibration range. For this reason, all indices are presented as estimates and are used for relative comparison between treatments rather than as absolute measures of nutritional value or animal performance. DMI is expressed as a percentage (%) of body weight (Table 7).

### 4.4. Statistical Analysis

The study data were subjected to statistical analysis based on a split-plot design within randomized complete blocks. Data from both years were evaluated using a combined analysis. Year, sowing density, and fertilization type were defined as fixed effects, while the block was included in the statistical model. The sub-plot was designated as the experimental unit, with one oilcake sample analyzed from each. Analytical replicates of the sampling process were not considered independent experimental units. A total of 64 samples were analyzed (*n* = 64). The study data were subjected to statistical analysis using the StatGraphics Centurion XVII v.17.2.00 statistical package (StatGraphics Technologies Inc., The Plains, VA, USA). The statistical package provided the sums of squares (SS), while appropriate error terms were used to calculate F-statistics: “year” was tested against the block(year) mean square; “D” (sowing density) and the “Y × D” interaction were tested against the whole-plot error [block(year) × D, df = 6]; and “F” (fertilization type), “Y × F”, “D × F”, and “Y × D × F” were tested against the sub-plot error (df = 36), in accordance with the literature [41]. Furthermore, residual normality was assessed using the Shapiro–Wilk test, and the homogeneity of variances was evaluated using Levene’s test. In cases where the above conditions were not met, the analysis was repeated after log-transforming the data; however, this transformation did not alter the statistical significance of the effects under study, and therefore the original values are presented in the results. Mean comparisons were performed using Tukey’s HSD method. For comparisons within the same density, the sub-plot error term was used, whereas for comparisons between densities, a composite error term was employed. The degrees of freedom for the composite error were calculated using the Satterthwaite method. In instances where only the fertilization factor was statistically significant, the means of the four fertilization types were compared using Tukey’s HSD test, based on the sub-plot error term. Since the year did not have a statistically significant effect on the results, the data are presented in an aggregated format covering both years of the study. Results are presented as mean ± SE, with a sample size of *n* = 8 for each density × fertilization combination, and *n* = 16 for each fertilization type (following the aggregation of both densities and both years). All comparisons were carried out at a 5% level of statistical significance (*p* ≤ 0.05).

## 5. Conclusions

Overall, the results of this study indicate that the type of fertilizer, and its interaction with sowing density, significantly influence both the yield and the quality of the oilcake. The use of urea with urease and nitrification inhibitors increased oilcake yield at the low density, whereas at the high density it performed similarly to the control, as fertilization reduced oilcake recovery. At the high sowing density, this treatment led to the highest protein and fat contents, although it also increased NDF. At the same time, organic fertilization, although it resulted in lower oilcake yield compared with urea with urease and nitrification inhibitors, significantly improved the protein content of the oilcake to levels similar to those of this treatment, with intermediate fiber contents. In conclusion, selecting the appropriate combination of sowing density and fertilizer type can modify both the productivity and the composition of the oilcake, although this involves trade-offs between oilcake yield and individual compositional traits. As the feeding indices were estimated using predictive equations, and the study was conducted with one cultivar at a single site over two growing seasons, further research is required before practical recommendations for its use as an animal feed product can be made.

## Figures and Tables

**Table 1:** Effect of sowing density and fertilizer type on oilcake’s dry matter, ash, protein and fat content.

	Dry Matter (DM) (%)		Ash (A) (%)		Crude Protein (CP) (%)		Fat (F) (%)	
	D1	D2	D1	D2	D1	D2	D1	D2
C	92.69 ± 0.06 ^b^	91.83 ± 0.07 ^a^	3.55 ± 0.02 ^a^	4.06 ± 0.01 ^d^	31.72 ± 0.11 ^a^	31.71 ± 0.29 ^a^	18.88 ± 0.05 ^c^	16.96 ± 0.08 ^a^
I	93.11 ± 0.05 ^d^	92.95 ± 0.04 ^cd^	4.23 ± 0.03 ^f^	4.29 ± 0.02 ^f^	35.38 ± 0.37 ^e^	33.63 ± 0.07 ^bc^	24.35 ± 0.07 ^f^	19.74 ± 0.03 ^d^
O	93.44 ± 0.02 ^e^	95.98 ± 0.03 ^f^	3.75 ± 0.01 ^c^	4.15 ± 0.01 ^e^	34.58 ± 0.11 ^de^	34.54 ± 0.12 ^cde^	21.91 ± 0.07 ^e^	17.74 ± 0.02 ^b^
U	92.87 ± 0.03 ^bc^	92.97 ± 0.03 ^cd^	3.83 ± 0.02 ^c^	3.64 ± 0.01 ^b^	33.77 ± 0.17 ^bcd^	33.27 ± 0.06 ^b^	19.88 ± 0.05 ^d^	17.73 ± 0.03 ^b^
F_YEAR(Y)_	3.13 ^ns^		2.00 ^ns^		0.03 ^ns^		2.71 ^ns^	
F_DENSITY(D)_	102.72 ***		251.96 ***		108.06 ***		75,484.70 ***	
F_FERTILIZER(F)_	1319.89 ***		369.47 ***		79.07 ***		2330.27 ***	
F_YXD_	0.35 ^ns^		2.23 ^ns^		0.01 ^ns^		0.45 ^ns^	
F_YXF_	1.66 ^ns^		0.16 ^ns^		0.03 ^ns^		1.62 ^ns^	
F_DXF_	666.72 ***		175.84 ***		7.52 ***		351.82 ***	
F_YXDXF_	0.65 ^ns^		0.08 ^ns^		0.35 ^ns^		1.82 ^ns^	

The mean values ± SE across two years (*n* = 8) of DM, A, CP and F are presented. Different letters within the columns indicate statistically significant differences among the eight density × fertilizer combinations (Tukey’s HSD, *p* < 0.05, split-plot error terms). Ash, crude protein and fat are expressed on a dry-matter basis. Non-statistically significant differences are symbolized using “ns”, while the symbol “***” indicate significance at the *p* < 0.001 levels.

**Table 2:** Effect of sowing density and fertilizer type on oilcake’s fiber content.

	Crude Fiber (Fi) (%)	
	D1	D2
C	7.16 ± 0.03 ^b^	7.21 ± 0.004 ^b^
I	7.89 ± 0.004 ^d^	7.89 ± 0.10 ^cd^
O	7.86 ± 0.03 ^cd^	7.02 ± 0.004 ^ab^
U	6.93 ± 0.01 ^a^	7.70 ± 0.04 ^c^
F_YEAR(Y)_	0.72 ^ns^	
F_DENSITY(D)_	0.05 ^ns^	
F_FERTILIZER(F)_	99.14 ***	
F_YXD_	0.46 ^ns^	
F_YXF_	0.79 ^ns^	
F_DXF_	115.42 ***	
F_YXDXF_	0.79 ^ns^	

The mean values ± SE across two years (*n* = 8) of Fi are presented in the present table. Different letters within the columns indicate statistically significant differences among the eight density × fertilizer combinations (Tukey’s HSD, *p* < 0.05, split-plot error terms). Crude fiber is expressed on a dry-matter basis, at *p* = 5% significance level. Non-statistically significant differences are symbolized using “ns”, while the symbol “***” indicate significance at the *p* < 0.001 levels.

**Table 3:** Estimated metabolizable energy (ME) for ruminants and pigs and total digestible nutrient (TDN) of flaxseed oilcake as affected by sowing density and fertilizer type.

		Metabolizable Energy (ME) (kcal/kg DM)		Total Digestible Nutrient (TDN) (%)
		Ruminants	Pigs	
D1	C	2856 ± 1 ^bc^	4478 ± 6 ^c^	78.99 ± 0.02 ^bc^
	I	2859 ± 2 ^c^	4656 ± 5 ^e^	79.08 ± 0.06 ^c^
	O	2855 ± 1 ^abc^	4605 ± 11 ^d^	78.96 ± 0.03 ^abc^
	U	2875 ± 1 ^d^	4416 ± 5 ^b^	79.51 ± 0.03 ^d^
D2	C	2855 ± 2 ^abc^	4378 ± 7 ^b^	78.96 ± 0.05 ^abc^
	I	2848 ± 3 ^a^	4406 ± 5 ^b^	78.78 ± 0.07 ^a^
	O	2877 ± 1 ^d^	4382 ± 10 ^b^	79.57 ± 0.02 ^d^
	U	2851 ± 1 ^ab^	4296 ± 13 ^a^	78.86 ± 0.04 ^ab^
F_YEAR(Y)_		0.04 ^ns^	1.02 ^ns^	0.04 ^ns^
F_DENSITY(D)_		23.27 **	4811.70 ***	23.27 **
F_FERTILIZER(F)_		25.02 ***	124.09 ***	25.02 ***
F_YXD_		0.32 ^ns^	0.03 ^ns^	0.32 ^ns^
F_YXF_		0.32 ^ns^	0.60 ^ns^	0.32 ^ns^
F_DXF_		67.29 ***	28.78 ***	67.29 ***
F_YXDXF_		0.37 ^ns^	0.14 ^ns^	0.37 ^ns^

Values are means ± SE across two years (*n* = 8). Different letters within the columns indicate statistically significant differences among the eight density × fertilizer combinations (Tukey’s HSD, *p* < 0.05, split-plot error terms). D1: 500 plants m^−2^; D2: 300 plants m^−2^; C: control; U: urea; I: urea with urease and nitrification inhibitors; O: organic fertilizer. Non-statistically significant differences are symbolized using “ns”, while the symbols “**” and “***” indicate significance at the *p* < 0.01 and *p* < 0.001 levels, respectively.

**Table 4:** Effect of factors on estimated DMI, DDM and RFV.

	Dry Matter Intake (DMI) (% of Body Weight)	Digestible Dry Matter (DDM)	Relative Feed Value (RFV)
C	7.55 ± 0.06 ^c^	81.74 ± 0.05 ^b^	478.1 ± 3.8 ^c^
I	6.01 ± 0.03 ^a^	81.40 ± 0.04 ^a^	379.3 ± 2.1 ^a^
O	7.00 ± 0.10 ^b^	81.54 ± 0.03 ^a^	442.6 ± 6.5 ^b^
U	6.01 ± 0.07 ^a^	81.41 ± 0.05 ^a^	379.6 ± 4.4 ^a^
F_YEAR(Y)_	0.18 ^ns^	1.32 ^ns^	0.21 ^ns^
F_DENSITY(D)_	0.01 ^ns^	4.61 ^ns^	0.04 ^ns^
F_FERTILIZER(F)_	80.67 ***	11.47 ***	82.10 ***
F_YXD_	0.01 ^ns^	0.01 ^ns^	0.01 ^ns^
F_YXF_	0.34 ^ns^	0.37 ^ns^	0.32 ^ns^
F_DXF_	0.04 ^ns^	1.35 ^ns^	0.03 ^ns^
F_YXDXF_	0.19 ^ns^	0.52 ^ns^	0.19 ^ns^

Values are means ± SE across sowing densities and years (*n* = 16). Different letters within the columns indicate statistically significant differences among fertilizer types (Tukey’s HSD, *p* < 0.05). C: control; U: urea; I: urea with urease and nitrification inhibitors; O: organic fertilizer. Non-statistically significant differences are symbolized using “ns”, while the symbol “***” indicate significance at the *p* < 0.001 level.

**Table 5:** The composition of fertilizers used and the application rate.

Treatment	Fertilizer	Composition	Producer	Application Rate (kg ha^−1^)	N Rate (kg ha^−1^)
C	Control	No fertilizer	-	–	0
U	Urea	46-0-0	EUROCHEM HELLAS S.A. Athens. Greece	174	80
I	Inhibited urea	Urea (46-0-0) + urease inhibitor (2-NPT 0.035%) + nitrification inhibitor (MPA 0.07%)		174	80
O	Compost + tomato pomace	Processing tomato pomace (50%) + plant-residue compost (50%; wheat and maize straw); 44% organic matter, 28.7 g kg^−1^ total N, 15 mg kg^−1^ Olsen P, 30 mg kg^−1^ K, EC 1.76 mS cm^−1^, pH 7.38 [20]	-	2784	80

**Table 6:** Measurements used in order to assess the quality of oilcake.

Parameter	Method	Instrument	Calculation	Reference
Dry matter (DM)	AOAC 934.01	Drying oven (103 °C)	Direct determination	[35]
Ash (A)	AOAC 942.05	Muffle furnace		
Fat (F)	AOAC 920.39	Soxtec 2055 (FOSS, Hillerød, Denmark); petroleum ether extraction		
Crude fiber (Fi)	AOCS Ba 6a-05	ANKOM 200 Fiber Analyzer (ANKOM Technology, Macedon, NY, USA)		[36]
Neutral detergent fiber (NDF)	Van Soest et al., without α-amylase, expressed exclusive of residual ash			[37]
Acid detergent fiber (ADF)	AOAC 973.18			[35]
Crude protein (CP)	AOAC 984.13	Vapodest auto-analyzer (C. Gerhardt GmbH & Co. KG. Königswinter. Germany)	Kjeldahl N × 6.25	

**Table 7:** Indicators used for animal feed quality per animal category.

Animal Category	Parameter	Equation	Unit	Reference
Pigs	Metabolizable energy (ME)	ME = 3931 + (53.77 × F) − (29.31 × NDF)		[30]
Ruminants	Total Digestible Nutrient (TDN)	TDN = 78.9 + (0.17 × CP) − (0.74 × Fi)	%	[31]
	Metabolizable energy (ME)	ME = 0.82 × 0.04409 × TDN × 1000	kcal kg DM^−1^	[40]
	Dry Matter Intake (DMI)	DMI = 120/NDF	% of body weight	[38]
	Digestible Dry Matter (DDM)	DDM = 88.9 − (0.779 × ADF)	%	[39]
	Relative Feed Value (RFV)	RFV = (DMI × DDM)/1.29	–	

## Data Availability

The data presented in this study are available on reasonable request from the corresponding author.

## Abbreviations

The following abbreviations are used in this manuscript:

ALA	A-linolenic acid
Y	Year
D1	High density (500 plants m^−2^)
D2	Low density (300 plants m^−2^)
C	Control
I	Inhibited urea
O	Organic fertilizer
U	Urea
DM	Dry matter
A	Ash
CP	Crude protein
F	Fat
Fi	Crude fiber
NDF	Neutral detergent fiber
ADF	Acid detergent fiber
ME	Metabolizable energy
TDN	Total digestible nutrient
DMI	Dry matter intake
DDM	Digestible dry matter
RFV	Relative feed value
SOM	Soil organic matter
CaCO_3_	Calcium carbonate
P	Phosphorus
K	Potassium
